# Sterol methyltransferase a target for anti-amoeba therapy: towards transition state analog and suicide substrate drug design

**DOI:** 10.1194/jlr.M079418

**Published:** 2017-10-17

**Authors:** Medhanie E. Kidane, Boden H. Vanderloop, Wenxu Zhou, Crista D. Thomas, Emilio Ramos, Ujjal Singha, Minu Chaudhuri, W. David Nes

**Affiliations:** Department of Chemistry and Biochemistry,* Texas Tech University, Lubbock, TX 79409; Department of Microbiology and Immunology,† Meharry Medical College, Nashville, TN 37208

**Keywords:** phytosterol biosynthesis, sterol C24-methyltransferase, ergosterol biosynthesis inhibitors, Acanthamoeba, anti-amoeba drugs

## Abstract

Ergosterol biosynthesis pathways essential to pathogenic protozoa growth and absent from the human host offer new chokepoint targets. Here, we present characterization and cell-based interference of Acanthamoeba spp sterol 24-/28-methylases (SMTs) that catalyze the committed step in C_28_- and C_29_-sterol synthesis. Intriguingly, our kinetic analyses suggest that 24-SMT prefers plant cycloartenol whereas 28-SMT prefers 24(28)-methylene lophenol in similar fashion to the substrate preferences of land plant SMT1 and SMT2. Transition state analog-24(*R,S*),25-epiminolanosterol (EL) and suicide substrate 26,27-dehydrolanosterol (DHL) differentially inhibited trophozoite growth with IC_50_ values of 7 nM and 6 µM, respectively, and EL yielded 20-fold higher activity than reference drug voriconazole. Against either SMT assayed with native substrate, EL exhibited tight binding ∼*K*_i_ 9 nM. Alternatively, DHL is methylated at C26 by 24-SMT that thereby, generates intermediates that complex and inactivate the enzyme, whereas DHL is not productively bound to 28-SMT. Steroidal inhibitors had no effect on human epithelial kidney cell growth or cholesterol biosynthesis at minimum amoebicidal concentrations. We hypothesize the selective inhibition of Acanthamoeba by steroidal inhibitors representing distinct chemotypes may be an efficient strategy for the development of promising compounds to combat amoeba diseases.

Human infections with amoeba represent rare diseases, yet they are an emerging health problem that can spread in unconventional ways, causing quick death, as in cases involving the spinal cord or amoeba that cross the blood-brain barrier (1). In contrast to flagellate protozoan parasites, such as the trypanosomes, that can have a complex life cycle including two morphologically different stages and using insects as vector and a variety of mammalian hosts, the Acanthamoeba life cycle proceeds simply from a trophozoite (cell) to cyst phase, with the trophozoite infecting and spreading in human hosts (2, 3). Current treatment for Acanthamoeba infections responsible for blinding keratitis or granulomatous amebic encephalitis (GAE) rely on an investigational alkylphosphocholine drug: milfetosine, biguanide, or the anti-fungal medical azole voriconaole (4–6). Against the paucity of treatment possibilities and understanding resistance to these drugs is on the rise, investigators have turned to sequencing the amoeba genomes to evoke a broad search for new, protozoan-specific drug targets (7, 8). Indeed, a BLAST search in the database of the Acanthamoeba genome revealed two sterol C24-methyltransferase (SMT) sequences as a highly promising potential drug target because SMT is synthesized in the parasite and not in the host organism. Involvement of SMT biosynthetically in amoeba is observed in the synthesis of C_28_-ergosterol and its C_29_-homolog 7-dehydroporiferasterol, final products in *Acanthamoeba spp* (9–12) (**Fig. 1**). In protozoa, the SMT-catalyzed reaction yields C24-methyl sterol product that is metabolized to ergosterol essential for growth (13, 14), which is the basis for SMT being an effective drug target.

**Fig. 1.**
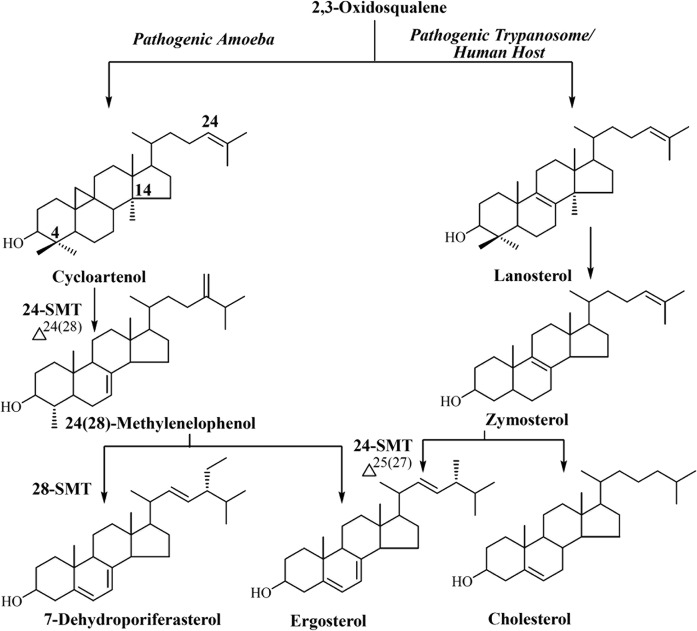
Metabolic pathways for the conversion of phyla-specific protosterol to C_28_-ergosterol, C_29_-7-dehydroporiferasterol and C_27_-cholesterol in pathogenic protozoa and human host; SMT, sterol methyltransferase.

Recent studies have focused on steroidal inhibitors of sterol methylation in ergosterol biosynthesis as potential assassins of pathogenic protozoa. We have reported that transition state analogs containing charged sulfonium or ammonium groups as mimics of the C24- or C25-carbocationic intermediates generated during trypanosome sterol methylations are tight binding inhibitors of SMT while substrate analogs that bear a reactive warhead have been developed as suicide inhibitors to covalently bind and inactivate the enzyme (13, 15–17). In order to assess the value of steroidal inhibitors of the sterol methylation reaction as anti-amoeba agents, we have begun to study the effects of such compounds on growth and sterol biosynthesis and to characterize the cDNA and recombinant SMTs and inhibitory profile studies in *Acanthamoeba castellinii* (Ac). Quite unexpectedly, in contrast to *Trypanosoma brucei*, which synthesizes a single SMT that converts substrate to Δ^25(27)^-C_28_- product, Ac synthesizes two SMTs that individually catalyze the formation of Δ^24(28)^-C_28_- and Δ^24(28)^- and Δ^25(27)^-C_29_-products in the amoeba. We determined the *Ac* 24SMT recognizes as a natural substrate cycloartenol whereas *Ac*28SMT recognizes 24(28)-methylene lophenol as a natural substrate, as is the case for the plant 24SMT and 28SMT (18, 19), respectively, whereas the favored substrate for *Tb*24-SMT is zymosterol, as is the case for fungal *Candida* and *Saccharomyces* (15, 20, 21). These enzymic differences noted for the first time across kingdoms in unikont (amoeba) and bikont (kinetoplastid) protozoan ergosterol biosynthesis afford the opportunity for enzyme- and parasite-specific suicide inhibitor recognition. Notably, the substrate preferences for the AcSMTs agree with the sterol metabolome characterization showing Ac operates a cycloartenol-based ergosterol pathway in similar fashion to *Dictyostelium* amoeba (22), which differs from trypanosomal protozoa that synthesize a lanosterol-based ergosterol pathway (23, 24). We now report an evaluation of steroidal inhibitors of differing mechanisms of action can be as effective at killing Ac trophozoites as medical azoles or moreso, that they are more effective against amoeba than trypanosomes or pathogenic fungi where suicide inhibitors are not permeable to the cell wall, and that the amoebicidal effects of these drugs are associated with the nature of the inhibitor-SMT complementation. These results provide insights into catalytic mechanism-based design of selective inhibitors, which would not interfere with cholesterol biosynthesis in the human host and would create opportunities for active-site labeling to enhance ergosterol biosynthesis sensitivity to combination therapies, of interest in the context of new leads to treat Acanthamoeba disease.

## MATERIALS AND METHODS

### Strain, culture conditions, and MAC determination

Ac strain ATCC 30010 was inoculated (1 × 10^4^ cells/ml) into tissue culture T-25 ml flasks, prepared with ATTC media 712 and cultured axenically in 5 ml medium at 25°C. Continuous cultures populated by trophozoites (<90%; the remaining cells were cysts) was maintained by sub-culturing 4–5 day growth arrested cells (1 × 10^6^ cells/ml) into fresh medium. Steroidal inhibitor and azole susceptibility assays were performed in 24-well (4 × 6) microtiter plates (3 ml total). Trophozoite assay conditions and preparation of stock solutions made to 1 μg/10 μl and 1 μg/100 μl in DMSO through serial dilution to achieve final inhibitor concentrations of 64, 32, 16, 8, 4, 2, 1, 0.5, 0.25, 0.125, 0.0625, and 0.03125 µg/ml in 1% DMSO as reported previously for voriconazole incubated with Ac (25). Growth curves to evaluate treated trophozoites were initiated with 1 × 10^6^ cells/ml and incubated for 48 h. Cell number was calculated with the use of a hemocytometer. The percentage of viable trophozoites following exposure to different concentrations of inhibitors was determined by the standard trypan blue exclusion method. Cells stained blue were considered nonviable. Treated cultures showing no viable cells routinely contained a few cysts (∼10^2^ to 10^3^ cysts/ml). The IC_50_ of inhibitors against trophozoite growth was evaluated using GraphPad Prism with default setting (GraphPad Software Inc., CA). The drug concentration responsible for minimum amoebicidal activity (MAC) was defined as the lowest concentration of inhibitor with no visible live trophozoites as determined by light microscopic inspection of treated cultures following trypan blue staining and microscope examination, which confirmed the cell death. Compounds were tested in triplicate (SD not greater than ± 10%) at each concentration.

### SMT gene identification, cloning, and expression

Sequence data for the Ac ORF was from the NCBI (https://www.ncbi.nlm.nih.gov/) website. A tblastn search was carried out using *Saccharomyces cerevisase* SMT (NCB accession number AAB31378) as the query sequence. Alignment was performed by Clustal W2.0 embedded with the Genious (9.1.8) software with BLOSUM matrix using a set of SMTs previously characterized by us that represent SMTs across kingdoms (15, 18–21, 26). Two putative SMT genes in Acanthamoeba were identified as SMT1 and SMT2; the gene identifiers of SMT1 and SMT2 in the GeneBank were introduced by the Strasbourg group for land plant SMTs capable of catalyzing the introduction of a methyl group from S-adenosyl-L-methionine (SAM) at C24 and C28, respectively (27). The Acanthamoeba genes (24-SMT, XP_004336540 and 28-SMT, XP_004335307) were synthesized by Eurofin MWG (Huntsville, AL) incorporating an Nbe1 restriction site at the 5′end and a BAMH1 restriction site at the 3′ end of the open reading frame, typical of our previous constructions of recombinant SMTs. Gene integrity was verified by PCR using gene-specific primers and by DNA sequencing. For confirmation of SMT overexpression, *Escherechia coli* BL21 transformed cells were selected on Luria agar plates containing ampicillin (100 μg/ml). Positive clones were verified by isolation of plasmid DNA, followed by restriction digestion and sequencing.

Phylogenetic analyses were performed by comparing the AcSMT amino acid sequence to selected SMT proteins across kingdoms from the NCBI data base with a phylogenetic tree created using Genious with the embedded FreeTree (2.1.5) program with default settings; the numbers of branches are substitutions per site. The SMT sequences used to evaluate the protein evolution across kingdoms were *A. castellanii-1* (XP_004335307), *A. castellanii-2* (XP_004336540), *Leishmania mexicana* (XP_003874589), *Clavispora lusitaniae* (XP_002617229), *Monosiga brevicollis* (XP_001748534), *Chlamydomonas reinhardtii* (XP_001690775), *Trypanosoma brucei* (XP_822930), *Yarrowia lipolytica* (XP_505173), *Glycine max-1* (NP_001238391), *Zea mays-*1 (ACG33830), *Z. mays*-2 (NP_001149131), *Trypanosoma cruzi* (EKG01467), *Candida albicans* (EEQ44277), *Paracoccidioides brasiliensis* (EEH21414), *G. max-2* (ACS93764), *Arabidopsis thaliana*-1 (AAM53553), *A. thaliana-*2 (AAM91592) and *S.cerevisiae* (AAB31378).

### Protein expression and enzyme assay

The SMT constructs were transformed into competent BL21(DE3) cells and transformants selected against 100 µg/ml ampicillin. Expression of the SMT constructs and partial purification of the recombinant proteins followed the procedure described previously (19). Briefly, a single colony for 24-SMT and 28-SMT were grown overnight in an orbital shaker (250–300 rpm) at 37°C in Luria broth containing ampicillin and then reinoculated in fresh medium at 1 in 10 dilution. Cells were allowed to grow for 1 to 2 h until the optical density (OD) at 600 nm was reached at 0.3 to 0.5 and divided into two tubes. In one tube, cells were induced by addition of isopropyl thio-galactoside at a final concentration of 1 mM and in the other tube, were un-induced. Cells were harvested 2 and 4 h after induction and an equal number of cells (as calculated by OD @600 nm) from induced (+) and un-induced (−) cultures were boiled with 1X Laemmli’s buffer and analyzed by SDS-PAGE (12%). Gel was stained with Coomassie Brilliant Blue (0.2%) to establish the molecular weight and purity of the expressed protein. Total protein concentrations were determined by Bradford dye-binding procedure with bovine γ-globulin as standard. To determine the catalytic activity of the recombinant proteins, enzyme assays were carried out using soluble bacterial extract (100,000 g fraction) in 9 ml test tubes containing a total of 600 µl assay volume for 45 min (initial velocity conditions). The assay buffer contained 20 mM phosphate buffer in 5% (v/v) glycerol at pH 7.5, sterol acceptor emulsified in 12 µl Tween 80 (1.2 g/l), and the substrate tested over the concentration range 5 to 150 µM against 1.2 mg of total protein. The reaction was initiated by the addition of saturating levels of [*methy*l-^3^H_3_]SAM, 0.9 µCi, at 150 µM. Incubation mixtures were saponified with methanolic potassium hydroxide and extracted with hexane (3 × 1 ml) and concentrated to dryness providing the nonsaponifiable lipid fraction; radioactivity was determined by liquid scintillation counting. For every substrate, the kinetic parameters of the reaction (*K*_mapp_ and *V*_maxapp_) determined under initial velocity conditions were calculated by fitting the liquid scintillation data to the Michaelis-Menten equation using the computer program GraphPad (7.0). Enzyme activity measurements were reproducible with an SE of ± 10%. Controls containing radioactive SAM and no sterol used to background subtract radioactivity in these treatments was less than 500 dpm.

For product analysis by GC/MS, the assay conditions were modified to contain saturating levels of 100 µM sterol acceptor and 1.2 or 2.4 mg total protein and the incubations carried out overnight. In control *E. coli* extracts, no contaminant sterol was detected by GC/MS (consistent with the prokaryote nature of *E. coli*) Product yields determined by GC were accurate to ∼1% (50 ng) and as necessary to detect lower yields selected ion monitoring of relevant ion clusters was employed.

Determination of inhibitor *K*_i_ was established from dose-response plots yielding IC_50_ values for the effects of steroidal inhibitor on the maximal velocity of 24-SMT or 28-SMT at saturating concentrations of sterol substrate (100 µM) and SAM (150 µM) against varied concentration of 24(*R,S*),25-epiminolanosterol varied from 0, 3.9, 15.6, 62.5, 250, to 1000 nM or 26,27-dehydrolanosterol varied from 0. 7.8, 15.6, 31.3, 62.5, 125 to 250 µM concentrations carried out using standard assay conditions. Assays were performed in triplicate with less than 10% deviation. Conversion of IC_50_ value to *K*_i_ as dissociation constant, based on the experimentally deduced kinetic properties of the native substrate and steroidal inhibitor against 24-SMT and 28-SMT, was accomplished using the Cheng-Prussoff equation (28).

### Inactivation studies

To show time-dependent inactivation, experiments were performed with a 100,000 g supernatant fraction containing the recombinant *Ac*24-SMT in two major steps. First, a preincubation experiment was established; to a 9 ml test tube containing DHL at the test concentrations (varied between 0 and 20 µM), 2.4 mg of total protein (ca. 160 µg SMT), 1.5 ml buffer, and a fixed concentration of SAM (100 µM) were added. The incubation was allowed to incubate for up to 20 min at 35°C. Second, at the time indicated, aliquots (20 µl) of the preincubation preparation were placed in a precooled (dry ice-ethanol) test tube to prevent catalysis. The frozen samples were then individually thawed and filtered three times with phosphate buffer to remove excess unbound inhibitor using Amicon ultracentrifugation filters. The remaining enzyme activity in each sample was determined by assaying each at saturating cycloartenol concentration (100 µM) and [*methyl*-^3^H_3_] SAM (100 µM; 0.6 µC) under standard assay conditions for 1 h. The logarithmic percentage of remaining enzyme activity was plotted against the incubation time of the enzyme-inhibitor mixture to determine the half-life (*T*_1/2_) of inactivation. A Kitz and Wilson plot was then generated using *T*_1/2_ at each inhibitor concentration and the inverse of inhibitor concentration on GraphPad (7.0) according to the equation –*T*/_1/2_ = 0.69/*k*_inact_ + 0.69/*k*_inact_ × *K*_i_/I where *k*_inact_ is the rate of inactivation and *K*_i_ is the concentration of inactivator that produces a half maximal rate at inactivation. The point of intersection with the ordinate is the *T*_1/2_ at saturation from which the *k*_inact_ can be calculated whereas the intercept along the X-axis gives the −1/*K*_i_ from which the *K*_i_ can be calculated.

For 24-SMT labeling, partially pure recombinant enzyme obtained from 0.25 salt gradient fraction after Q-chromatography and desalted with buffer exchange (as reported in reference 19) was generated as a reference specimen of cloned SMT migrating at ∼39 kDa indicated by a protein ladder (Bio-Rad). Protein was stained with Coomassie Brilliant Blue R 250. To determine covalent binding, 1.2 mg of a soluble protein (100,000 g supernatant) was incubated with 100 µM DHL and [^3^H_3_-*methyl*]SAM (1.1 × 10^6^ dpm) commixed with carrier SAM to 150 µM and assayed for 1 h at 35°C. After filtration and concentration, 22 µg total protein was loaded on 12% SDS-PAGE gel against a protein ladder (Bio-Rad) that ranged from 15 to 100 kDa. Next, five bands were excised from the gel corresponding to approximate molecular masses that range between *1*) 15–25 kDa, *2*) 25–35 KDa, *3*) 35–45 kDa, *4*) 45–55 kDa, and *5*) 55–75 kDa, placed into a vial of 50% hydrogen peroxide, and left overnight at 60°C for digestion (29). To these vials was added scintillant and the radioactivity (^3^H in dpm) measured by liquid scintillation counting.

### Chemical and instrumental analysis

The source of sterol substrates evaluated in this study is described in our earlier papers (18–21). 24(*R,S*),25-epiminolanosterol (EL) and 26,27-dehydrolanosterol were prepared as previously reported (30, 31). Voriconazole was purchased from Sigma. All sterols were purified by HPLC to >95% by GC analysis. SAM chloride salt was purchased from Sigma. [^3^H_3_-*methyl*]SAM (specific activity 10–15 Ci/mMol, and diluted to 10 µCi/µMol) was purchased from S/D/N isotopes (Pointe-Claire, QC) and [^2^H_3_-*methyl*]SAM (99.3% ^2^H enrichment) was purchased from MSD isotopes (Candada). The Bradford protein assay kit was purchased from Bio-Rad and isopropyl-1-thio-β-D-galactoside was purchased from Research Products International Corp. All other reagents and chemicals were from Sigma or Fisher unless otherwise noted.

Instrumental methods have been reported previously (18–21). Briefly, mass spectra were obtained on a Hewlet-Packard 6890 GC-HP 5973 MSD instrument (electron impact, 70 eV, scan range 50–550 amu). Capillary GC (0.25 mm i.d. by 30 m fused silica column coated with Zebron ZB-5 (Phenomenex) was operated at a flow rate of helium set at 1.2 ml/min, injector port at 250°C, and temperature program of initial 170°C, held for 1 min, and increased at 20°C/min to 280°C. GC analysis of sterols is reported as RRTc values referring to the retention time of sample GC peak to retention time of cholesterol peak which moved typically in the chromatogram at 13.8 min or slightly longer to 14.5 min depending on whether the column tip was clipped due to age issues. In sterol analysis, product distributions were determined by approximate integration of chromatographic peaks. HPLC was carried out as described (26).

### Cell metabolite identification

Amoeba and HEK cells were harvested at one or more points during growth in the presence and absence of steroidal inhibitor and azole at IC_50_ or MACs of the inhibitor or at 40 µM for HEK cells. Cell pellets were split with an internal standard of known amounts of 5α-cholestane added to one of the cell pellets for determination of sterol amounts in cells. Cells were saponified directly in aqueous methanolic potassium hydroxide at the reflux temperature for 1 h, which yielded total sterol (free plus sterol released from ester bonds). The resulting neutral lipids obtained by dilution with water and extraction with hexane (Fisher) were analyzed by GC/MS or HPLC-equipped with a diode array detector against cholesterol as a chromatographic reference. GC and HPLC retention times of sterols were compared with those obtained from authentic standards in our steroid collection (18–22). To determine the trophozoite sterol metabolome, GC/MS analysis of cell pellet nonsaponfiables was typically carried out using control cultures incubated in 5 ml medium in 25 ml T-flasks inoculated with 1 × 10^4^ trophozoites/ml and cultured for 72 h. To generate significant cell pellets for sterol analysis of treated cells, trophozoites were cultured in 10 ml medium in 25 ml T-flasks inoculated with 1 × 10^5^ cells/ml and grown for 48 h.

## RESULTS

### Trophozoite phytosterols

Ac trophozoites have been reported to synthesize two main phytosterols, ergosterol and 7-dehydroporiferasterol in a 5 to 7 ratio (9), otherwise the sterol composition for this *Acanthamoeba* species is unknown. Using GC/MS/HPLC analysis, we confirmed Ac trophozoites harvested at the onset of stationary phase growth generated ergosterol and 7-dehydroporiferasterol in a 5 to 7 ratio, which represented 95% total sterol (**Table 1**). However, in contrast to the Smith and Korn (9) observations, we could identify several minor sterols in trophozoites. These compounds were similar to those identified previously in *A. polyphaga* (12), suggesting the sterol biosynthesis pathway is the same for *A. polyphaga* and Ac (Fig. 1). The sterol isolation approach used by Smith and Korn (9) in the 1960s to form steroidal-digitonides will not form complexes of C4-methyl sterol intermediates such as cycloartenol. However, cell saponification-extraction with hexanes can yield C4-methyl sterols; using this method, we observed trace to significant amounts of C4-methyl sterol intermediates in cells depending on the phase of growth, including cycloartenol and 24(28)-methylene cycloartanol, which accumulate to 30% total sterol in the lag to log stages of early growth. These observations indicate high SMT activity is important to Ac growth.

**TABLE 1.** Major sterols (% of total sterol determined by GC/MS) of growth-arrested trophozoite cells and human cells

Sterol	Organism*^a^*		
	Ac	Ap	HEK
Cholesterol (Δ^5^)			100
Ergosterol (Δ^5, 7, 22^)	40	42	
7-Dehydroporiferasterol(Δ^5,7,22^)	55	trace	
Poriferasterol (Δ^5,22^)	trace	5	
Δ^7^-Poriferasterol (Δ^7,22^)	trace	48	
Minor sterols	5*^b^*	5*^c^*	

a Ac, *Acanthamoeba castellanii* (this study; note the C_28_-sterol and C_29_-sterol compositions can change during trophozoite growth and encystment); Ap, *A. polyphaga* (10); HEK (this study), Human epithelial kidney cells.

b Poriferasterol,^2^Δ^7^-Poriferasterol,^2^Brassi-casterol, ^2,3^Cycloartenol,^2,3^24-Methylenecycloartanol, ^2,3^Cycloeucalenol, ^2,3^Obtusifoliol, ^2,3^ Ergost-8(14)-enol.

c 7-Dehydroporiferasterol (see Figs. 1 and 6 and ref. 10 for key to structures).

### Sequence alignment and phylogenetic analysis of *Ac*SMT

**Fig. 2.**
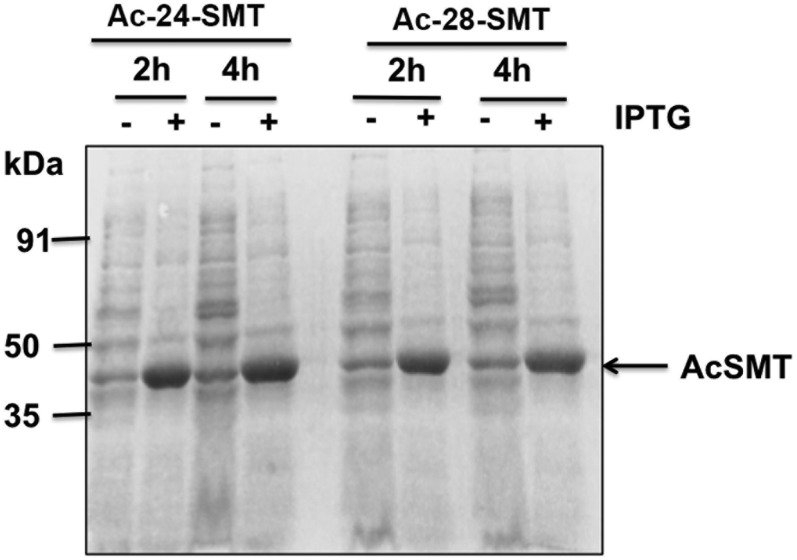
Comparison of amino acid sequences of sterol methyltransferases of *Acanthamoeba castellanii* with related enzymes across kingdoms. Cr, *Chlamydomonas reinhardtii* (green alga); Ac, *Acanthamoeba castellanii* (amoeba protozoa*)*; Gm, *Glycine max* (land plant); Tb, *Trypanosoma brucei* (kinetoplastid protozoa); Tc, *Trypanosoma cruzi* (kinetoplastid protozoa); and Sc, *Saccharomyces cerevisiae* (fungus). Sterol methylation carried out as the first (Δ^24(25)^-substrate) C_1_-transfer reaction and second (Δ^24(28)^-substrate) C_1_-transfer reaction is correlated to the corresponding 24-SMT (=SMT1) and 28-SMT (=SMT2) sequences detected in the Genebank. Substrate binding segments established in references 29, 36 for sterol (Regions I, III and IV) and SAM (Region II) are highlighted by black and blue boxes, respectively. Conserved residues are in red.

**Fig. 3.**
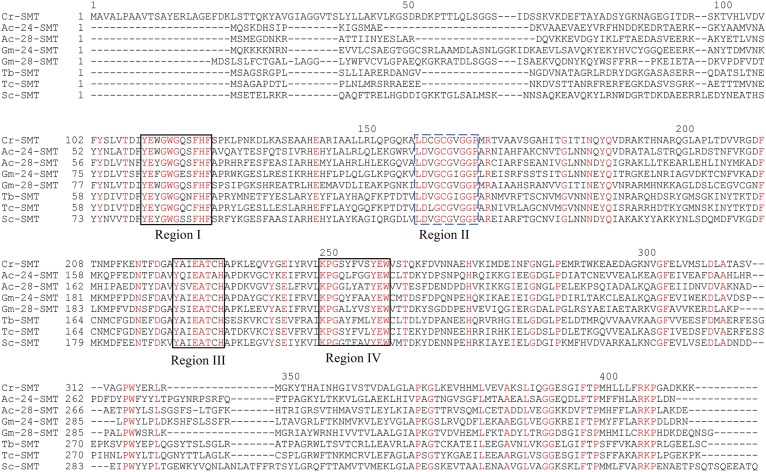
An amino acid-based phylogenetic tree of selected SMT enzymes across kingdoms associated with their substrate preference for a Δ^24(^25^)^ - sterol or Δ^24(^28^)^ -sterol. Putative *A. castellanii* 24-SMT and 28-SMT are in green labeled SMT1 and SMT2, respectively, as they are identified in the Genebank. The tree was constructed as described in Materials and Methods; substitution per site is labeled on the branch.

Given that the biosynthesis and architectural suitability of C_28_- and C_29_-phyotsterols in amoeba appears to be similar to land plants (12, 27), the question emerges whether SMT catalysis could be a chokepoint reaction [defined as a reaction that either consumes a unique substrate or produces a unique product (32)] and, thus, provide a model system for investigating the underlying enzymic sensitivities to steroidal inhibitors that can interfere with sterol C24-methylations in parasites. Also relevant to our studies is that SMTs are conserved by taxomomic origin rather than by biochemical function yielding specific products because either Δ^24(28)^ - or Δ^25(27)^ -sterol products formed by SMT can convert to cycloartenol- or lanosterol-based ergosterol and its C28-homolog, 7-dehydroporiferasterol (24, 33, 34). Therefore, we were interested first in identifying the endogenous SMTs from an amoeba-*Acanthamoeba* to complement the previously recognized SMTs in trypanosomes, fungi, and plants. Toward this end, by performing a BLAST search on the Ac genomic database, two SMT genes were identified. The corresponding encoded proteins (**Fig. 2**) were singled out as SMT1, or by our nomenclature, 24-SMT, of predicted Mr = 39,023 Da and SMT2, or 28-SMT, of predicted Mr = 39,257 Da. The two amino acid sequences are 70% similar and 34% identical to each other. Phylogenetic analysis reveals Ac 24-SMT shares higher sequence identity, ∼48% to land plant 24-SMT than to fungal 24-SMT ∼39% identity, whereas *Ac*28-SMT sequence shows moderate identity ∼34% to the known plant 28-SMT, suggesting *Ac*24-SMT and *Ac*28-SMT should possess specific plant-like substrate requirements as proposed by Raederstorff and Rohmer (12) on chemical reasonableness. A phylogenetic tree constructed with the *Ac*SMT genes against related SMT genes across kingdoms indicated further that amoeba SMTs are more related to plant SMTs than to protozoa SMTs (**Fig. 3**). The *Ac*SMTs loosely resemble land plant SMTs, each possessing clustered regions in their alignment of significant homology arranged in the same order and separated by comparable intervals along the polypeptide chain of SMTs. Figure 2 shows four conserved motifs, three complement sterols (Regions I, III and IV, 35, 36), and one complement SAM (Region II, 29). Regions I to IV are highly homologous, possessing ≥80% sequence identity among the enzymes for these sites. Region I, which stretches from Tyr81 to Phe-91 (Erg6p numbering system) and is enriched with aromatic residues, is shown by affinity labeling and mutagenesis to direct the methylation-deprotonation reaction of cation (high energy intermediate)- π (aromatic residue) stabilizations responsible for product diversity unique to sterol methylations (35, 36).

### *Ac*SMT expression and characterization

**Fig. 4.**
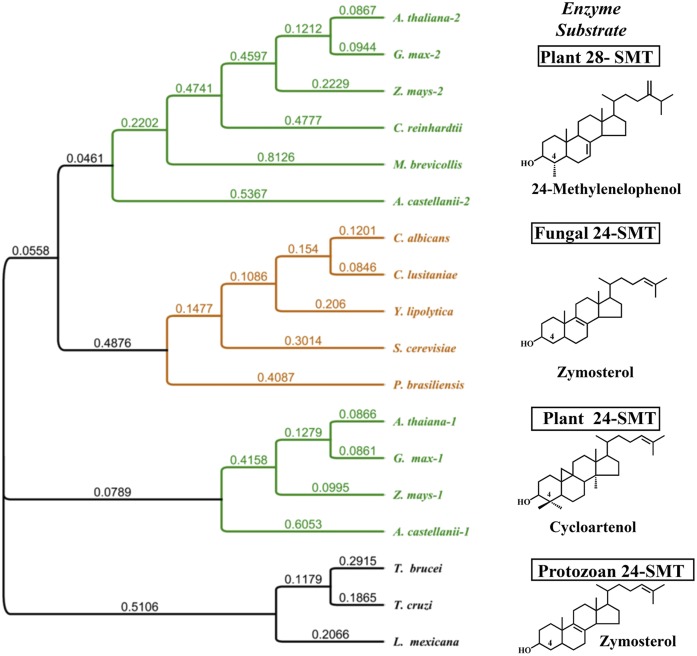
Expression of *A. castellani* 24-SMT (SMT1) and 28-SMT (SMT2) in *E. coli*. *Ac*SMT1 and *Ac*SMT2 genes were cloned in pET11a expression plasmid as described in the Materials and Methods and transformed in competent *E. coli* BL21(DE3) cells. Gel was stained by Coomassie Brilliant Blue (0.2%) to visualize the protein bands. Molecular weight markers are shown. The position of the recombinant protein is indicated by an arrow. IPTG, isopropyl thio-galactoside.

Using *E. coli* BL21 lysate preparations of uninduced SMT assayed with saturating cycloartenol or 24(28)-methylene lophenol paired with SAM yielded unchanged sterol substrate in the reacted enzyme extract. Alternatively when the *E. coli* BL21 cells harboring *Ac*SMT gene was activated, target protein was evident migrating on SDS-PAGE with the predicted size of ∼39 kDa (**Fig. 4**). To examine the properties of the two *Ac*SMTs, a soluble enzyme extract was prepared by methods previously employed in the study of protozoa SMT cloned and expressed in bacterial cells that do not synthesize sterols. Although we observed increasing enrichment of *Ac*SMT during purification by SDS-PAGE (data not shown), differential loss of SMT activity as noted in previous studies in SMT purification can occur, yielding homogenous SMT with a low turnover number of ∼0.6 min^−1^ (15, 16, 19). Recently, we determined the reason for major loss of activity during protein purification is attributable to the allosteric nature of SMT (homotetramer showing cooperativity among its subunits), which contains a Walker motif for ATP binding in the SMT sequence. During SMT purification through chromatography, ATP, established as a positive allosteric effector of SMT activity, is removed from the protein such that the resulting pure SMT is minimally active, converting substrate to product in 5–20% yields depending on the individual SMT (unpublished observations). Therefore, product analysis and kinetic evaluation were performed on lysate or soluble preparations, which were highly active as determined by GC/MS analysis. These preparations contained ∼80 µg of active SMT determined by the stoichiometric complex of DHL with *Ac*24-SMT established by the concentration of C26-methyl DHL diol generated in the enzyme extract and using SMT as a tetramer for the native subunit organization in the calculations of turnover number.

**Fig. 5.**
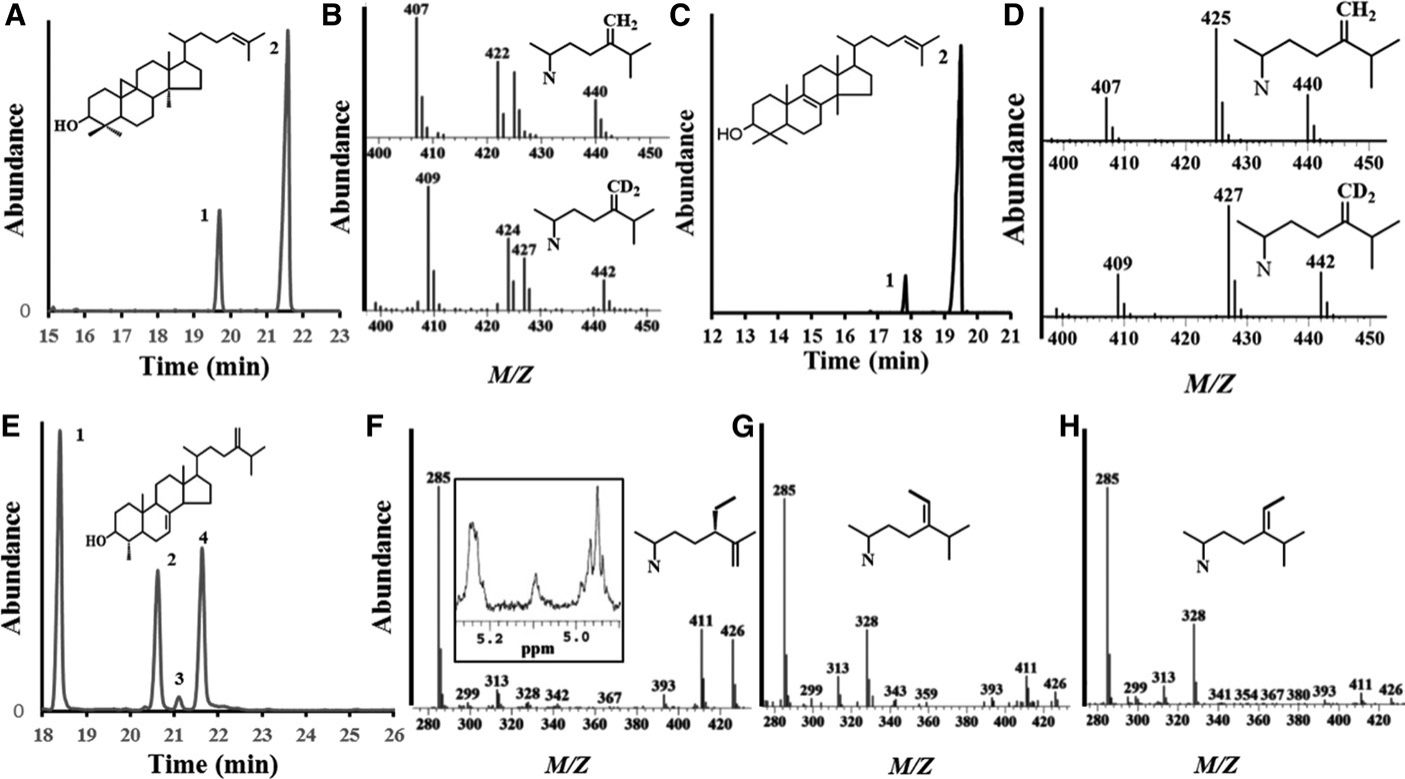
GC/MS analysis of *Ac*24-SMT generation of single product and *Ac*28-SMT generation of multiple products. A: cycloartenol (pk-1, substrate) conversion to 24(28)-methylene cycloartanol or D_2_-24(28)-methylenecyloartanol (pk-2) by 24-SMT. B: mass spectra of products for SAM (top) and [^2^H_3_-*methyl*]SAM (bottom) incubations with cycloartenol yielding pk-2, top. C: lanosterol (pk-1, substrate) conversion to eburicol (pk-2) by 24-SMT. D: mass spectra of products for SAM (top) and [^2^H_3_-*methyl*]SAM incubations with lanosterol yielding pk-2, (D). E: 24(28)-methylenelophenol (pk-1, substrate) conversion to 24β-ethyl 4α-methyl stigmast-7, 25(27)-dienol (pk-2), 4α-methyl stigmast-7, 24(28)*E*-ethylidene dienol (pk-3) and 4α-methyl stigmasta 7, 24(28)Z-ethylidine dienol (pk-4). F: mass spectrum of sterol in pk-2 (E); inset is the olefinic region in ^1^HNMR of the sample recovered from HPLC and containing trace 24(28)Z-ethylidene isomer. G: mass spectrum of sterol in pk-2. H: mass spectrum of sterol in pk-4. Incubations were carried out overnight with sterol substrate and SAM at saturation as described in Materials and Methods.

Enzyme preparations were assayed initially with substrates considered to be native to Ac-cycloartenol for 24-SMT and 24(28)-methylenelophenol for *Ac*28SMT and product outcomes determined using GC/MS analysis of the enzyme-generated organic extracts. The reaction products generated by expression of the two *Ac*SMTs were substrate-dependent, with enzyme-specific activities similar to land plant 24-SMT and 28-SMT (18, 19). Comparison of the RRT_c_ and MS data with available synthetic standards unambiguously identified the enzymatic products as 24(28)-methylene cycloartanol (GC peak 2) generated by 24-SMT and 24β-ethyl 4α-methyl stigmasta-7,25(27)-dienol (GC peak 2), 4α methyl stigmasta-7, 24(28)*E*-ethylidine-dienol (GC peak 3), and 4α methyl stigmasta 7,24(28)*Z*-ethylidine-dienol (GC peak 4) generated by 28-SMT (**Fig. 5**). Further confirmation for the Δ^24(28)^ -olefin structure for cycloartenol conversion product is shown in the incubation with [^2^H_3_-*methyl*]SAM, which generated a C28 methyl group of two mass unit increases (M^+^ 442) compared with incubation with SAM as shown in Fig. 5A. Preparative-scale 28-SMT assay followed by HPLC of the enzyme-extract to fractionate the products provided sufficient material for 500 MHz ^1^HNMR of the phytosterol corresponding to pk-2 contaminated with some P-4 material (Fig. 5E); the olefinic region in the ^1^HNMR of this sample (referenced to deuterated chloroform) possessed chemical shifts at 5.239, brs for H7 and at 4.811, d, 2.2 Hz for H25 for major HPLC isolate and at 4.949 dt 3.9/1.5 Hz for H28(Z-geometry) of contaminant isolate in HPLC fraction (26, 37). The triplet of *Ac*28-SMT generated products, also detected in land plant SMT catalysis (35), result from partitioning of a common intermediate during the sterol 28-methylation reaction, with product diversity of 24β-ethyl 4α-methyl stigmasta-7,25(27)-dienol from loss of H27 proton and 4α methyl stigmasta-7, 24(28)*E*-ethylidine-dienol and 4α methyl stigmasta 7,24(28)*Z*-ethylidine-dienol from loss of H28 at the *E* and *Z*-positions (19) (Fig. 5). It is noteworthy that *Ac*24-SMT catalyzes the regiospecific introduction of the exocyclic methylene group at C24(28) with similar fidelity to the land plant 24-SMTs.

The catalytic competence (*k*_cat_/*K*_m_) for 24-SMT and 28-SMT against cycloartenol and 24(28)-methylenelophenol, respectively, was estimated to be about 1.5 min^−1^/ 44 µM/ and 0.8 min^−1^/25 µM/ from Lineweaver-Burk plots of the kinetic data. The two AcSMTs exhibit slow-acting catalysis and a similar catalytic competence of ∼0.03 (M^−1^ s^−1^). The conversion rates were linear with increasing time up to 90 min in a total soluble protein concentration of 2 mg/ml. It is of interest and of probable physiological significance as to whether 24α-methyl/ethyl or 24β-methyl/ethyl sterols are final products, that the *Ac*28-SMT activity generates turnover rates similar to land plant 28-SMT (18). Notably, Ac28-SMT differs from land plant 28-SMT in the pattern of product distribution that contain a higher proportion of 4α methyl stigmasta-7,24(28)*Z*-ethylidene dienol (>90% of total product in the 24-ethyl sterol mixture) important to C_29_-intermediate formation involved in sitosterol synthesis which at C24 is stereochemically opposite to 7-dehydroporiferaterol bearing an α-ethyl group compared with a β-ethyl group, respectively.

### Substrate specificities of AcSMTs

**Fig. 6.**
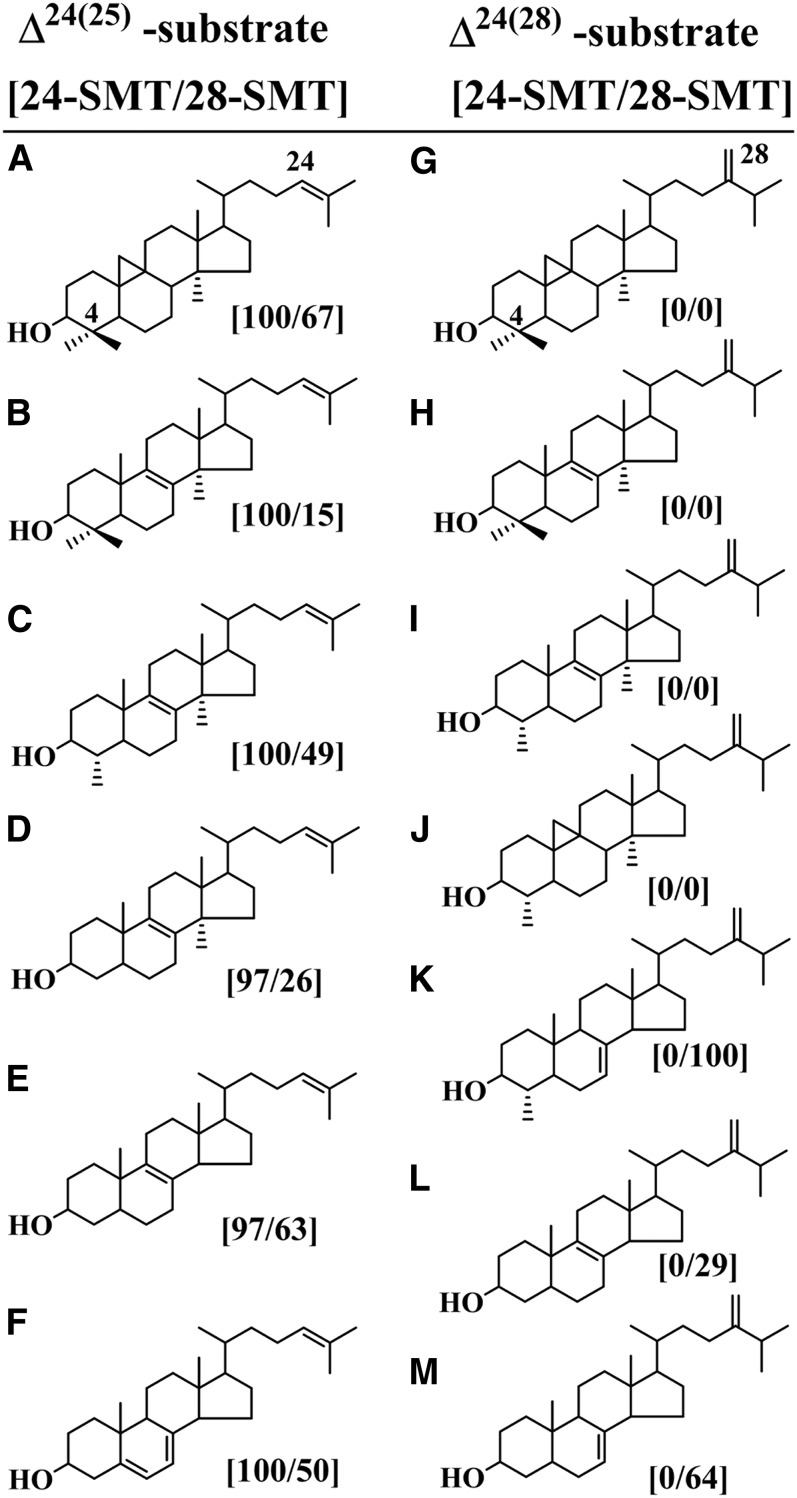
Product ratios generated by Ac24-SMT and Ac28-SMT incubated with either a Δ^24(^25^)^ - or Δ^24(^28^)^ -sterol substrate. The product ratios are normalized to cycloartenol at ∼70% conversion rates. Sterols are: (A), cycloartenol, (B), lanosterol, (C), 31-norlanosterol, (D), 14α-methylzymosterol, (E), zymosterol, (F), cholesta-5,7,24-trienol, (G) 24(28)-methylenecycloartanol, (H) eburicol, (I), obtusifoliol, (J), cycloeucalenol, (K), 24(28)-methylenelophenol, (L), fecosterol and (M), episterol.

To test whether the active sites of 24-SMT and 28-SMT can accommodate alternate Δ^24(25)^ - or Δ^24(28)^ -substrates, various substrates were chosen for their natural occurrence in protozoa and product percentages by GC/MS analysis were evaluated with the two SMTs at saturating levels of substrate incubated for 45 min at 35°C (Fig. 5). Comparing the substrate binding determinants of A to M in Fig. 5 that underlie the regiochemistry of the product distributions generated by 24-SMT and 28-SMT shows a broad substrate range for C24-methylation, whereas specific substrates are required for C28-methylation. SMT favors the 4,4-dimethyl cycloartenol whereas the 28-SMT favors the C4-monomethyl 24(28)-methylenelophenol. Substrates having a C24(28)-olefin group showed no productive binding to 24-SMT whereas those with a C24(25)-olefin group were accepted differentially by either 24-SMT or 28-SMT. GC/MS analysis of product formations showed no change in the product ratios compared with incubations with cycloartenol or Δ^24(28)^-methylenelophenol, shown in Fig. 4. In general, 28-SMT showed better binding to Δ^24(28)^ -methylene lophenol than to eburicol, obtusifoliol, 24(28)-methylenecycloartenol, cycloeucalenol (differentially missing C4β-methyl group, Δ^8^-bond, 14α-methyl group or 9,19-cyclopropyl group), fecosterol, or episterol (missing C4-methyl groups) (**Fig. 6**). The presence of a 9,19-cyclopropyl group, which does not alter the pseudo-flat conformation of 9,19-cyclopropyl-compounds as once proposed (38), does not preclude productive binding in the case of lanosterol, which can convert to the same product set generated by cycloartenol incubated with 24-SMT or 28-SMT. Comparison of C24(25)-olefinic substrates with C24(28)-olefinic substrates against the two SMTs shows strict specificity in the case of 28-SMT, which emphasizes the order of intermediates reported in Fig. 1 for ergosterol biosynthesis. Recognizing the importance of disrupting carbon flux and 14-methyl substrate homeostasis in ergosterol biosynthesis, we moved next to show the ability to block substrate binding using different inhibitor chemotypes can inhibit SMT activities and in so doing, interfere with trophozoite viability.

### Inhibition of SMT by substrate mimics

**Fig. 7.**
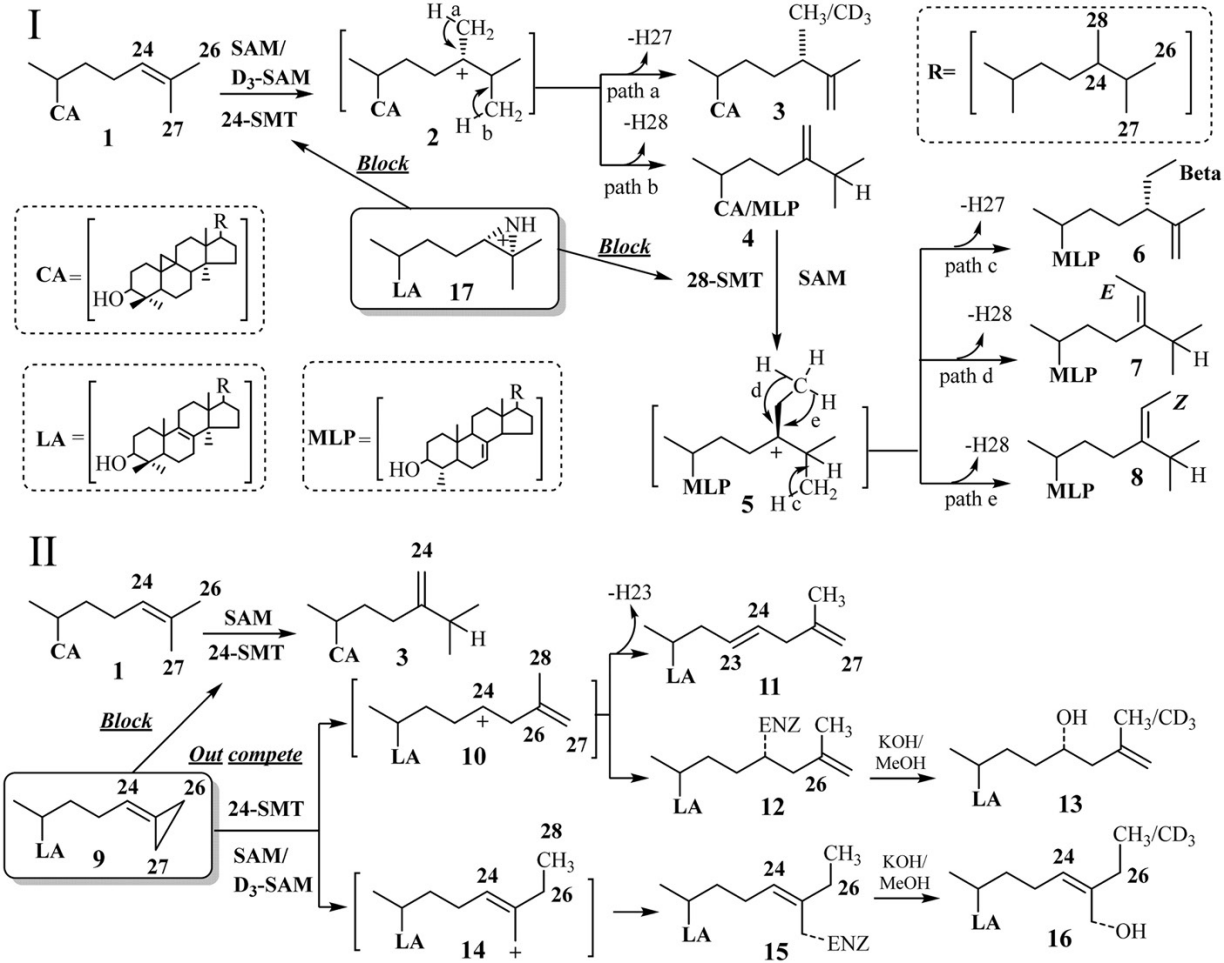
Sterol methylation pathways for the conversion of Δ^24(25)^ - or Δ^24(28)^ -substrate to Δ^24(28)^ - or Δ^25(^27^)^ -olefin products, indicating possible hydride shift and deprotonation mechanisms along with proposed site of inhibition by 24(*R,S*),25-epiminolanosterol (I) and mechanism of enzyme inactivation by 26,27-dehydrolanosterol (II).

**Fig. 8.**
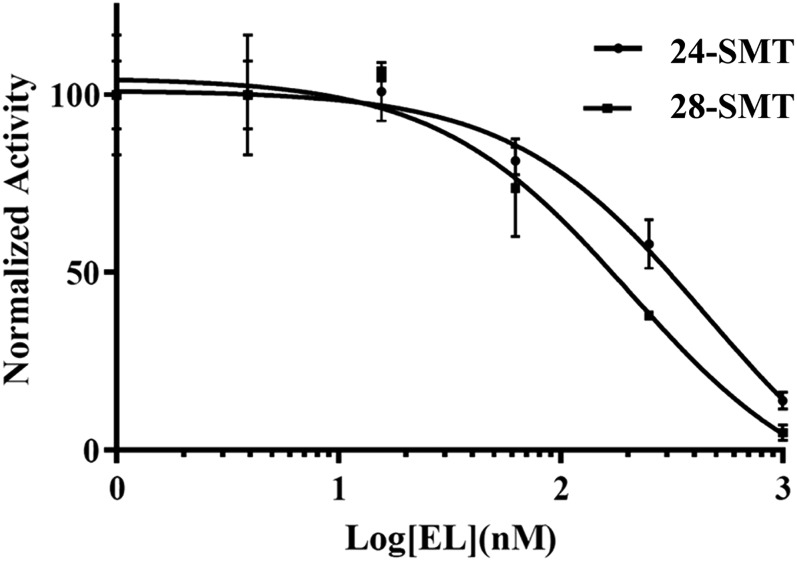
Inhibition of *Ac*24-SMT and *Ac*28-SMT by 24(*R,S*),25-epiminolanosterol yielding IC_50_ values of 44 nM ± 4 nM and 20 nM ± 2 nM, respectively; *K*_i_ values of steroidal inhibitor were determined from the corresponding IC_50_ value as detailed in Materials and Methods. Normalized activity is related to control incubations for 100% activity. Incubations were carried out under initial velocity conditions of 45 min with sterol (100 µM) and [^3^H_3_-*methyl*]SAM (150 µM). For control, ^3^H-methyl product afforded ∼1 × 10^6^ dpm for substrate cycloartenol or 5 × 10^5^ dpm for substrate 24(28)methylene lophenol whereas assays without sterol yield ∼500 dpm. Error bars represent ± SEM of three independent experiments.

**Fig. 9.**
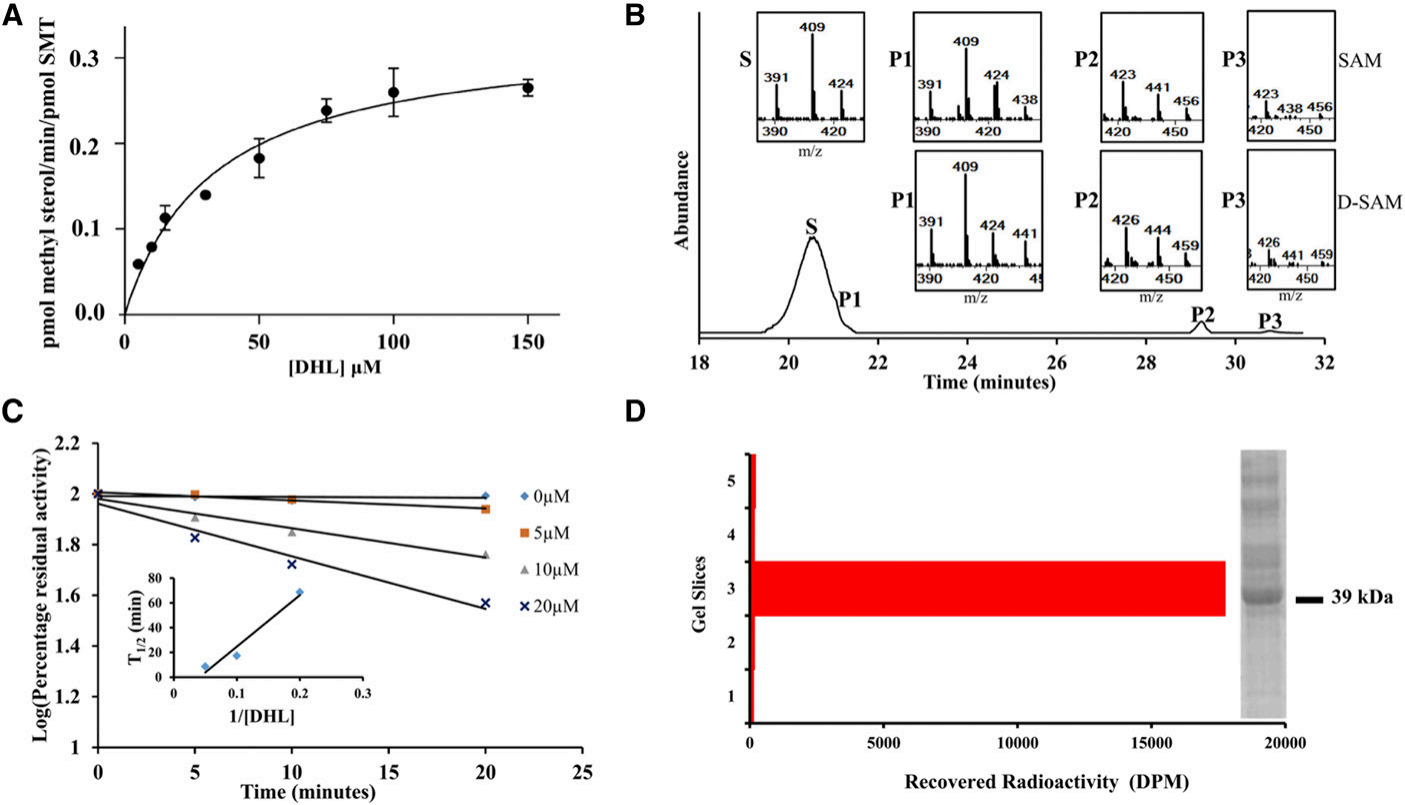
Kinetic and chemical analysis of *Ac*24-SMT activity from incubation with 26,27-dehydrolanosterol (DHL). A: K_m_ curve of DHL paired with saturating levels of 150 µM SAM assayed under initial velocity conditions described in Materials and Methods. B: GC/MS analysis of enzyme-generated products recovered from nonsaponifiable lipid fraction of assay with saturating cosubstrates at 2.4 mg total protein. C: Inactivation of 24-SMT with the mechanism-based inhibitor in the concentration range and scheduled time of incubation as shown; inset: plot of 1/*k*_inact_ plotted as T_1/2_ (min) versus 1/inhibitor. Specific labeling of *Ac*24-SMT with DHL. D: The Coomassie-stained SDS-PAGE gel of the partially pure cloned enzyme referenced to Bio-Rad ladder 20 to 100 kDa aligned next to the gel of DHL C26-methyl metabolite-complexed with 24-SMT. The position of 24-SMT is shown by predicted molecular mass associated with the 40 kDa marker.

It has been shown previously that 24(*R,S*),25-epiminolanosterol (EL = **15**, **Fig. 7**), an analog of the high energy intermediate, namely, a transition state analog formed by methylation of the terminal carbon = carbon bond of zymosterol or cycloartenol (Fig. 7), is a potent inhibitor of the 24-SMT activity in fungi, protozoa, and cultured plant cells (20, 34, 39). Related electronic mimics of the high energy intermediates with positive charges in the side chains located at positions resembling C24 or C25 cations show equal potency against 28-SMT (13, 20). Thus, the ability of EL to inhibit *Ac*24-SMT and *Ac*28-SMT activity was investigated here, with an observed *K*_i_ (based on the IC_50_ curves in **Fig. 8**) of 14 and 4 nM, respectively, similar to that reported against fungal, protozoan, and land plant SMTs (20, 34, 39). Alternatively, the potentially more reactive analog 26,27-dehydrolanosterol (DHL = **9**, Fig. 7) proved to be less effective as a competitive inhibitor against 24-SMT and 28-SMT, assayed with their natural substrate exhibiting *K*_i_s of 16 and 54 µM, respectively (data not shown). The measured *K*_i_ of DHL against *Ac*24-SMT was comparable to the Michaelis constant for *Ac*24-SMT of 44 µM assayed with cycloartenol, indicating DHL to be an effective substrate mimic of the natural substrate. In addition, the low *K_i_* value and competitive nature of the compound in assays with SMT is consistent with the rational design of DHL as a suicide substrate (17). *Ac*24-SMT incubated with [*methyl-^3^H_3_*]SAM and DHL generated a binding constant and turnover rate of ∼33 µM and 0.03 min^−1^, respectively (**Fig. 9**), whereas no activity was observed when the cosubstrates were incubated with *Ac*28-SMT.

### Chemical properties of DHL products

GC/MS analysis of the products resulting from incubation of DHL with *Ac*24-SMT showed poor conversion, indicating the fused C26-C27-methyl group interferes with optimal binding. The terminal methylene cyclopropane ring on DHL, which binds differently to SMT than the terminal isopropyl group on lanosterol, has sufficient nucleophilic character at C26 for methylation to occur at this carbon rather than at C24. The analog converted to a major (P1, 3%) product, *m/z* 438 (plus D_3_-SAM = *m/z* 441) and two minor (P2, 1%; P3,0.2%) products, *m/z* 456 (plus D_3_-SAM = 459), identical in GC retention time and mass spectrum with 26(28)-methyl lanosta-8(9), 23(24), 26(27)- trienol (C26-monol), 26(28)-methyl 24-hydroxy lanost-8(9), 25(27)-dienol (C26-diol), and 26(28)-methyl 26-hydroxy lanosta-8(9), 24(25)-dienol (C26-diol) obtained from incubation of DHL with a fungal SMT (40). The 3-deuterium atom incorporation into the P2 and P3 diols agreed with methyl addition at C26 rather than C24 and partitioning generates two intermediate cations that can be quenched at the active site. Alternatively, no methyl products were detected from the DHL incubation with 28-SMT consistent with the weak binding of lanosterol by 28-SMT.

### Mechanism of suicide inhibition

To explore the mechanism of DHL inhibition, we carried out incubations of *Ac*24-SMT with DHL and analyzed for suicide inhibition by GC/MS analysis and kinetically. Preliminary experiments using the same enzyme preparation split into two samples demonstrated that treatment of the enzyme-generated extract with chloroform-methanol (method 1) led to detection of substrate and P1 product whereas saponification of the enzyme preparation followed by extraction with hexane (method 2) led to substrate and P1, P2, and P3 products. Base generated release of such steroidal diols indicates there is an ester bond involved in the covalent linkage between the C26-methyl intermediate and an acidic residue in the active site of the enzyme. Thus, the presence of steroidal diols (C3OH and side chain OH) in the nonsaponifible lipid fraction is a chemical signature for the corresponding C26-methyl intermediates covalently attached to the enzyme (35). Because the inhibitor binds stoichiometrically to SMT (tetramer with one active site, 41), it is possible to calculate the amount of active SMT against the amount of C26-methyl diol formed. Thus, in 1.2 mg total protein, we estimate the amount of active SMT is ∼80 µg/512 pmol, which is less than 10% of the total protein in the soluble preparation. As anticipated for an active site process, in no case was inhibition observed when SAM was omitted from an otherwise complete reaction mixture and there was linear formation of steroidal diol formation and loss of enzyme activity after DHL incubation with 24-SMT. A replot of the inactiviation data as log (residual activity) against time for the three inhibitor concentrations gave a series of straight lines (Fig. 9C) showing that the inactivation followed pseudo-first-order kinetics under these conditions. A plot of the reciprocal of *k*_inact_, determined from the half lives of inactivation, versus the reciprocal of analog concentration (Fig. 9C), gave for the intercept a maximum rate of inactivation of ∼0.4 min^−1^ and T_1/2_ of 17 min. The partition ratio (*k*_cat_/*k*_inact_), a measure of formation of C26 methyl monol product per inactivation event, generated by 24-SMT incubated with DHL is ∼1, which is close to the partition ratio of 3 for 26-flourocholesta-5,7,24-trieniol against *Tb*SMT (16), suggesting DHL is a potent inactivator of *Ac*SMT activity. Selectivity of the time-dependent nature of covalent inhibitors such as DHL with their target proteins is often measured as *k*_inact_/*K*_i_ values in preference to IC_50_ values (12). In keeping with this recommendation, we observed a *k*_inact_/*K*_i_ value for DHL against *Ac*24-SMT of 2.5 (M^−1^ s^−1^), whereas the value is effectively zero for DHL against 28-SMT.

To examine covalent catalysis with a nucleophile from the analog itself, partially pure cloned 24-SMT was treated with DHL and ^3^H-SAM under standard assay conditions for 45 min at 35°C, and the resulting protein-inhibitor complex was analyzed for tritium by counting radioactivity in gel slices removed from the SDS-PAGE gel (Fig. 9D). The majority of the radioactivity (<90%) was associated with the protein band of molecular mass ∼39 kDa, which corresponds to the molecular mass and predicted migration of cloned 24-SMT. These results are entirely consistent with the proposed requirement for the analog to bind to the functional active site and undergo the methylation and deprotonation steps of the catalytic cycle in order for protein alkylation to occur.

### Inhibition of Acanthamoeba trophozoite growth and sterol biosynthesis

**Fig. 10.**
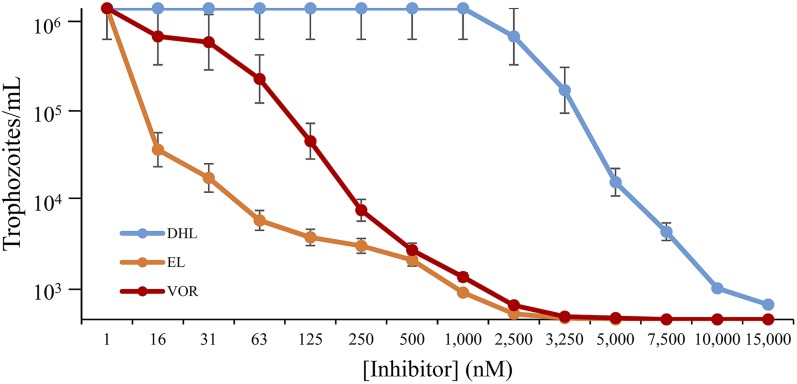
Differential effect of steroidal inhibitors and reference voriconazole on Ac trophozoite growth. DHL, 26,27-dehydrolanosteol, El, 24(R,S),25-epiminolanosterol, VOR, voriconazole. The SEMs are less than 5% of three independent experiments.

The effects of EL and DHL on trophozoite growth were compared against voriconazole found previously to be the most effective azole of a range of medical azoles to inhibit Ac growth (25). In this study, the IC_50_ values for trophozoite growth inhibition by EL and DHL were 7 nM and 6 µM, respectively, compared with 140 nM for voriconazole (**Fig. 10**), which is in good agreement with our previously reported value of IC_50_ 390 nM for voriconazole (25). The complete inhibition of growth, or MAC, at 48 h incubation was achieved at ∼2 µM EL, 15 µM DHL, and 3 µM voriconazole. No inhibition of growth was observed to 40 µM when the inhibitors were incubated with HEK cells. The selective index (AC-MAC/HEK-40 µM) was estimated for EL, DHL, and voriconazole at 20, 3, and 13, respectively. Comparison of the IC_50_ growth values reveal tight binding inhibitors of sterol methylation to be more effective than the CYP51 inhibitor voriconazole while the suicide inhibitor of SMT is moderately active and within the acceptable MAC range to be considered a chemotherapeutic lead. The sterol compositions of trophozoite treated cells incubated with steroidal inhibitor or azole were different from previous effects of these compounds incubated with kinetoplastid protozoa where the substrate of the target enzyme accumulates to significant levels at IC_50_ of inhibitor (13, 34). Notably, for trophozoites treated with EL or voriconazole, the substrate of the target enzyme never accumulates more than 1% total to 48 h. In DHL treatment, inhibitor was detected in cells by GC/MS at ∼0.5% total sterol. However, no relevant C4-methyl sterol intermediate or DHL metabolite was evident by GC/MS analysis. The lack of accumulation of cycloartenol following in vivo treatments by DHL is probably a result of inactivation of the 24-SMT affecting the enzyme turnover which, like the tight binding EL, blocks 24-SMT action (16). In contrast to our observations of 26-fluorolanosterol and DHL incubations in *T. brucei* where the lanosterol-based inhibitor was not a suicide substrate but rather was converted to an acceptable zymosterol analog capable of SMT binding (and thus acted as prodrugs, 16, 17), 15 µM DHL-treated cells at 48 h harvest showed DHL (RRTc, 1.24, M^+^ 424) and 26-methyl DHL (RRTc, 1.26, M^+^438) at 1% total sterol in an ∼10:1 ratio; no C26-methyl diol (M^+^456) was present within our limits of detection in GC/MS analysis. Thus, in *Acanthamoeba*, DHL appears to act directly on 24-SMT without prior metabolism.

Steroidal inhibitors clearly disrupted sterol homeostasis in *Acanthamoeba* as the final 24-alkyl sterol product ratio changed soon after the onset of treatment. Thus, 1 µM EL-treated cells showed ∼20% reduction in 7-dehydroporiferasterol at 24 h (ergosterol to 7-dehyrodroporiferaterol ratio of 50:50) and at 48 h there was a 40% reduction in 7-dehydroporiferasterol (ergosterol to 7-dehydroporiferasterol ratio of 6:4). Alternatively, 15 µM DHL-treated cells showed minimal effect on sterol composition at 24 h while at conclusion of the 48 incubation, the C_28_- to C_29_-sterol balance changed markedly as well yielding an ergosterol to 7-dehydroporiferasterol ratio of 55:45. The novel and unexpected changes in phytosterol composition following treatment with SMT inhibitors indicate blockage of the first acting enzyme-24-SMT can be overcome by downstream 28-SMT, which can accept cycloartenol poorly. The effect of blocked 24-SMT is to redirect carbon flux yielding alternate Δ^24(25)^-substrates that can compete with 24(28)-methylene lophenol for 28-SMT binding, generating a slower rate of C_29_-turnover and an uncommon C_28_-product that converts to ergosterol. These disturbances in forward metabolism by altered *Ac*24-SMT and *Ac*28-SMT activities appear to be responsible for the change in sterol stability, including C_28_ to C_29_-sterol balance that interferes with *Acanthamoeba* growth. In land plants, the physiological importance of C_29_-to C_28_ phytosterol balance is well characterized (27). This is the first report to show a requirement for a specific C_29_- to C_28_-phytosterol balance in protozoa growth.

## DISCUSSION

**Scheme 1.**
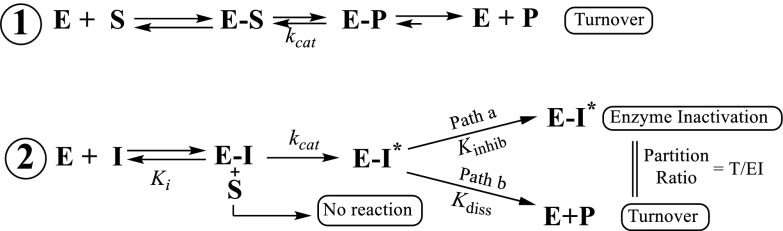
Kinetic schemes for *Ac*SMT inhibition by substrate analogs. In **1**, binding of enzyme (E) and Δ^24^-sterol substrate (S) followed by enzyme catalysis with formation of methyl product (P). In **2**, inhibitor (I) binds to enzyme followed by activation via methylation (E_ˑ_I*) and this intermediate can partition along path **b** and dissociate from the active site (E + P) or enter into path **a** and irreversibly bind to the enzyme (E−I*).

There are two basic inhibition approaches to killing an organism. Either the flux through an essential metabolic pathway is blocked, depressing final product biosynthesis to a level incapable of growth and sustaining life, or a biosynthetic intermediate can be increased to toxic levels. Although both of these are likely to involve enzyme inhibition, they are not the same, as observed in ergosterol biosynthesis where different inhibitor chemotypes, suicide inhibitor and tight binding inhibitor, have been studied against kinetoplastid protozoa (12). The former will normally require high affinity analogs of the natural substrate equipped with an electrophilic warhead of latent functionalities capable of complexing covalently with the target enzyme, whereas the latter is most effective as cationic substrates of the bound intermediate that can form an isomerized complex EI* of slow inhibitor release. The formation of isomerized EI* complexes generally correspond to situations where the inhibitor bears structural similarity to the transition state of enzymatic reaction or to high energy intermediate on the energy profile of the reaction, as in the case of sterol methylation inhibitors, or to charged interactions that stem from the metal ion-enzyme-inhibitor complex, as in azole inhibition of CYP51. Although a suicide substrate may be seemingly less effective as an anti-protozoan drug because its dosage to inhibit growth may be several orders of magnitude greater than a tight binding inhibitor, it may nonetheless possess significant chemotherapeutic benefit once the analog outcompetes the native substrate for binding to the SMT. For example, rationally designed analogs that structurally mimic the substrates of cholesterol biosynthesis could, without undergoing harmful metabolism, pass through the blood safely, and after relevant accumulation into the parasite, provide potential prevention of emergence of drug resistance due to continuous target suppression. The general strategy for inhibition of the parasite SMT can be illustrated as shown in **Scheme 1** where ES, EI, and ESI are the Michaelis complex (enzyme E bound with substrate S), the enzyme-competitive inhibitor complex, and the ternary complex resulting from binding of the inhibitor to the Michaelis complex, respectively.

Both irreversible and tight-binding steroidal inhibitors may be particularly suitable to direct SMT activity away from 24-alkyl sterol synthesis. We chose to evaluate our potential inhibitors as derivatives of lanosterol, because lanosterol is readily available commercially for synthetic purposes and at the start of these studies, lanosterol was shown to bind equally well as cycloartenol to 24-SMT. Recent studies (13, 20, 21, 40) with several SMT enzymes employing sulfonium ion and ammmoniun ion analogs of the reactive intermediates together with alternate substrates have suggested that these enzymes are not only capable of recognizing a broad range of steroidal inhibitors but also of converting those possessing rationally designed reactive functional groups to intermediates that can inactivate the enzyme. Sen­sitivity to sterol methylation inhibitors offers a unique opportunity for drug target identification and the subsequent development of new chemotypes as anti-amoeba agents.

In our design of steroidal inhibitors to prevent trophozoite growth, we considered the target enzyme should appear first in the 24-alkyl sterol biosynthesis pathway, must catalyze a nonequilibrium reaction, and that its activity controlled by substrate complementarity. In addition, it should be absent from animal host. Although not evident from the chemical characterizations, we observed Ac synthesized two SMT genes, *Ac*24-SMT and *Ac*28-SMT, that encode for functional enzymes of distinct substrate specificities and together act as rate-limiting enzymes that affect the pattern of sterol diversity in the amoeba. As shown in Fig. 2, there is considerable sequence similarity between the two *Ac*SMTs and between different SMTs across kingdoms. Heterologous expression of the *Ac*SMTs in *E. coli* resulted in the unusual ability for the product profile of the native SMTs to vary with the type of substrate used in vitro. In addition, our results show that *Ac*24-SMT and Ac 28-SMT are potently inhibited by EL whereas only the 24-SMT is inactivated by DHL, showing inhibitor specificity for the compounds tested. The tight binding inhibitor EL was found to have *K*_i_ values against the two SMTs of ∼9 nM, close to the IC_50_ value of 7 nM for growth inhibition. Alternatively, DHL was shown to be more than a competitive inhibitor; it is the first rationally designed enzyme-activated inhibitor of ergosterol biosynthesis in amoeba. DHL had a *K*_i_ value of 16 µM, a *k*_inact_ value of 0.4 min^−1^, and a *k*_cat_ value of 0.3 min^−1^. The data show for cholesta-5,7,22,24-tetraenol both concentration- and time-dependent inhibition for the active site of 24-SMT as well as an effective enzyme inactivator. The observed saturation kinetics indicate that a kinetic step subsequent to formation of the reversible *K*_m_ complex (E + I↔E·I), was rate-limiting. Time-dependent enzyme inactivation required SAM cofactor, indicating that DHL must be transformed by 24-SMT to a reactive intermediate before enzyme inactivation could occur in the active site (Scheme 1). The substantial rate-retarding effect on formation of methylated product measured as catalytic competence (*k*_ca_t/*K*_m_) by DHL is greater than 10-fold different from that of the natural substrate of 24-SMT, suggesting that steric perturbations by the fused methylene bridgehead across C26-C27 may alter the optimally oriented flexible side chain in the active site to favor a different partitioning directed at the methylated 26-carbon intermediate. The mechanism of DHL inhibition against 24-SMT is proposed to involve initial C-methylation at carbon-26, in keeping with the three D-atom incorporation into the sterol side chain from D_3_-SAM incubation to a pair of ring-opened intermediates (**10** and **14** in Fig. 7) that can be trapped by an active site nucleophile, resulting in irreversible inhibition of the enzyme. In the case of the corresponding analog 26,27-dehydrozymosterol incubated with [^3^H_3_-*methyl*]SAM and the yeast SMT (35, 41), the inhibitor was isolated as labeled methyl 26,27-dehydrozymosterol covalently bound to an acidic amino acid in a peptide fragment of trypsin-digested purified SMT, which confirms the current observation of DHL on Ac 24-SMT of irreversibility using cloned enzymes.

In conclusion, the results reported here indicate that Ac trophozoites were extremely sensitive to the effects of steroidal inhibitors. Irrespective of the inhibitor’s mechanism of action, using the 24-well cell-based system, these drugs generate cell death at MACs in the low micromolar range, which for EL is slightly below that of voriconazole, whereas they had no effect on cell growth of cultured human cells at similar concentrations to the MAC. The data show that these steroidal inhibitors limit cell growth of only the pathogenic protozoa and not cultured human cells as we observed previously in similar studies carried out on the bloodstream form of *T.* responsible for sleeping sickness (17). The in vivo IC_50_ values correspond closely to the in vitro IC_50_ values of the drugs against the isolated 24-SMT. Although the potency of the transition state analog EL and suicide inhibitor DHL on the growth response are vastly different, as reported before in *T. brucei* (13, 17), these differences are to be expected for mechanistic reasons. Thus, DHL targets enzyme inhibition through covalent binding suppressing the de novo synthesis of SMT, whereas the bound protonated EL suppresses the steady-state activity of the enzyme and then is released from the active site (19). The loss of biosynthetic capacity in treated trophozoites indicated by growth response to the mechanistically different steroidal inhibitors leads us to hypothesize that sterol methylation reactions and the balance of C_28_- to C_29_- sterol contribute to cell viability. For these reasons, SMT stands out as a new drug target in the pathogenic amoeba and the distinct steroidal chemotypes may thus be useful as leading compounds for the synthesis of selective inhibitors effective against Acanthamoeba diseases.
